# STIM1 as a key regulator for Ca^2+ ^homeostasis in skeletal-muscle development and function

**DOI:** 10.1186/2044-5040-1-16

**Published:** 2011-04-04

**Authors:** Santeri Kiviluoto, Jean-Paul Decuypere, Humbert De Smedt, Ludwig Missiaen, Jan B Parys, Geert Bultynck

**Affiliations:** 1 Laboratory of Molecular and Cellular Signaling, Department Molecular Cell Biology, K.U. Leuven, Campus Gasthuisberg O/N-1 bus 802, Herestraat 49, BE-3000 Leuven, Belgium

## Abstract

Stromal interaction molecules (STIM) were identified as the endoplasmic-reticulum (ER) Ca^2+ ^sensor controlling store-operated Ca^2+ ^entry (SOCE) and Ca^2+^-release-activated Ca^2+ ^(CRAC) channels in non-excitable cells. STIM proteins target Orai1-3, tetrameric Ca^2+^-permeable channels in the plasma membrane. Structure-function analysis revealed the molecular determinants and the key steps in the activation process of Orai by STIM. Recently, STIM1 was found to be expressed at high levels in skeletal muscle controlling muscle function and properties. Novel STIM targets besides Orai channels are emerging.

Here, we will focus on the role of STIM1 in skeletal-muscle structure, development and function. The molecular mechanism underpinning skeletal-muscle physiology points toward an essential role for STIM1-controlled SOCE to drive Ca^2+^/calcineurin/nuclear factor of activated T cells (NFAT)-dependent morphogenetic remodeling programs and to support adequate sarcoplasmic-reticulum (SR) Ca^2+^-store filling. Also in our hands, STIM1 is transiently up-regulated during the initial phase of *in vitro *myogenesis of C2C12 cells. The molecular targets of STIM1 in these cells likely involve Orai channels and canonical transient receptor potential (TRPC) channels TRPC1 and TRPC3. The fast kinetics of SOCE activation in skeletal muscle seem to depend on the triad-junction formation, favoring a pre-localization and/or pre-formation of STIM1-protein complexes with the plasma-membrane Ca^2+^-influx channels. Moreover, Orai1-mediated Ca^2+ ^influx seems to be essential for controlling the resting Ca^2+ ^concentration and for proper SR Ca^2+ ^filling. Hence, Ca^2+ ^influx through STIM1-dependent activation of SOCE from the T-tubule system may recycle extracellular Ca^2+ ^losses during muscle stimulation, thereby maintaining proper filling of the SR Ca^2+ ^stores and muscle function. Importantly, mouse models for dystrophic pathologies, like Duchenne muscular dystrophy, point towards an enhanced Ca^2+ ^influx through Orai1 and/or TRPC channels, leading to Ca^2+^-dependent apoptosis and muscle degeneration. In addition, human myopathies have been associated with dysfunctional SOCE. Immunodeficient patients harboring loss-of-function Orai1 mutations develop myopathies, while patients suffering from Duchenne muscular dystrophy display alterations in their Ca^2+^-handling proteins, including STIM proteins. In any case, the molecular determinants responsible for SOCE in human skeletal muscle and for dysregulated SOCE in patients of muscular dystrophy require further examination.

## Review

### STIM is the ER Ca^2+ ^sensor that controls Orai-mediated store-operated Ca^2+ ^influx

For about 20 years after the initial concept of store-operated Ca^2+ ^entry (SOCE) was proposed by Putney [1,2], the molecular candidates underpinning SOCE remained elusive. In 2005 and 2006, key players for SOCE in non-excitable cells were identified via RNAi screens in *Drosophila *[3] and HeLa cells [4], which elucidated STIM proteins as the endoplasmic-reticulum (ER) Ca^2+ ^sensor and Orai proteins [5-7] as the Ca^2+^-permeable store-operated Ca^2+ ^channel or Ca^2+^-release activated Ca^2+ ^(CRAC) channel [8,9]. In mammals, two STIM genes, *STIM1 *and *STIM2*, and three ORAI genes, *ORAI1*, *ORAI2 *and *ORAI3*, have been identified [5-7]. Different reports confirmed that STIM1 and Orai1 are the molecular candidates for currents with the electrophysiological properties of the CRAC channel [10-12]. These properties include a high selectivity for Ca^2+ ^over monovalent ions, like Na^+ ^and K^+^, and a single-channel Ca^2+ ^conductance of about 30 fS, which is about 100 times smaller than the conductance of L-type Ca^2+ ^channels [13,14].

STIM1 is predominantly located in the ER, the main intracellular Ca^2+ ^store [4,15-17]. The ER-resident STIM1 controls Orai function by activating these channels upon ER Ca^2+^-store depletion [3,12,17-19]. Recently, the different steps involved in the STIM1-dependent activation of Ca^2+ ^influx upon store depletion have been identified [15,16,20,21]. These steps include the sensing of Ca^2+ ^depletion from the ER, dissociation of Ca^2+ ^from the EF-hand motif of STIM1, the rapid oligomerization of STIM1, the translocation of STIM1 into punctae consisting of close ER/plasma-membrane junctions and the activation of plasma-membrane Ca^2+^-influx channels (see [13,14] for recent reviews).

STIM1 (approximately 75 kDa) contains an intraluminal region of approximately 22 kDa, a single transmembrane domain and a cytosolic region of approximately 51 kDa. The functional intraluminal ER Ca^2+ ^sensor of STIM1 is the first of two EF-hand domains (EF1; aa 63-96), which precedes a second EF-hand domain (EF2; aa 97-128) and a sterile α-motif domain (SAM; aa 131-200) [13,22]. The cytosolic domain consists of two or three coiled-coil domains within an ezrin-radixin moesin (ERM) domain, a serine/proline-rich (S/P) domain and a polybasic lysine-rich (KKK) domain. Structural analysis of the recombinantly expressed EF-SAM domain (aa 58-201) revealed that Ca^2+ ^is bound to the first EF-hand domain. EF-SAM exists in a monomeric state when Ca^2+ ^is bound because of close interaction of the paired EF hands and SAM [22,23]. When Ca^2+ ^dissociates, protein unfolding triggers major structural rearrangements of EF-SAM and the accumulation of dimer and aggregated forms of EF-SAM are observed [22,23]. *In vitro *experiments revealed a K_d _of about 500-600 μM for Ca^2+ ^binding to EF-SAM [23], which is in the range of Ca^2+ ^concentration ([Ca^2+^]) in the ER [21].

Ca^2+ ^dissociation from EF1 of STIM1 causes its oligomerization [22,23] and underpins the sequential changes upon ER Ca^2+^-store depletion [20]. Mutations in EF1 disrupting the Ca^2+^-binding properties of STIM1 or mutations in EF2 and SAM domain destabilizing the interaction between the EF-hand domains and SAM, result in a constitutively active STIM1 and activation of Orai proteins, resembling their state during depleted ER Ca^2+ ^stores [4,17]. Oligomerization of STIM1 is closely related to activation of CRAC, since artificial oligomerization of the STIM1 cytosolic domains was sufficient to trigger punctae formation and Ca^2+ ^influx [21]. These results indicate that the oligomerization of STIM1 is the switch that controls SOCE upon ER Ca^2+^-store depletion via STIM1/Orai1 clustering at ER/plasma-membrane junctions [21].

Upon STIM1 oligomerization, STIM1 redistributes to sites of close apposition of ER and plasma membrane [15,16,24]. The kinetics of STIM1 redistribution is in the order of tens of seconds and involves local diffusion in the ER membranes, while interaction with specific lipids or proteins may facilitate the accumulation of ER/plasma-membrane contact sites [4,16,20]. Two factors seem to contribute to STIM1 relocalization: i) protein-lipid interactions, mediated by the interaction of the polybasic C-terminus of STIM1 with plasmalemmal phospholipids like phosphatidylinositol 4,5-bisphosphate and phosphatidylinositol 3,4,5-trisphosphate, and ii) protein-protein interactions, mediated by the direct interaction of STIM1 with the C-terminus of Orai [20,25-28]. The latter interactions are proposed to contribute to the recruitment of Orai1 to ER/plasma-membrane junctions. Indeed, C-terminal truncation of Orai1 fails to co-localize with STIM1 punctae and hence fails to mediate CRAC currents upon store depletion [29,30]. A final step in the activation process is the opening of the tetrameric, Ca^2+^-selective Orai1 channels. *In vitro *Ca^2+^-flux assays revealed that Orai1 channels are directly gated by STIM1 [31]. The N-terminal cytosolic domain of Orai1 seems critical for Orai1-channel opening [30], possibly involving a direct binding of STIM1 to aa 65-91 of Orai1 [26,29,31]. Recently, the minimal region of STIM1 involved in CRAC activation was identified as the CRAC-activating domain (CAD, aa 342-448), also known as the STIM1-Orai1-activating region (SOAR, aa 344-442) [26,32-34].

Very recently, Orai1 channels were shown to be directly activated by the SPCA2, a Ca^2+ ^pump belonging to the secretory-pathway Ca^2+ ^ATPases [35]. SPCA2 expression potentiated Ca^2+ ^influx through Orai1 channels, independently of STIM proteins or SPCA2 Ca^2+^-ATPase activity. The mechanism involves a two-step activation mechanism and interaction of two parts of SPCA2: binding of the N-terminal region of SPCA2 to Orai1 enables SPCA2's C-terminus to access and activate Orai1 [35]. These findings are clinically relevant, since SPCA2 is up-regulated in breast tumors and SPCA2 knockdown decreases tumorigenicity.

STIM2 is very similar to STIM1 in basic structure and functional properties [4,36]. STIM2 senses luminal ER Ca^2+ ^via two EF-hands. Ca^2+ ^dissociation from STIM2 leads to a conformational change, oligomerization and redistribution to ER/plasma-membrane contact sites [4]. The redistribution of STIM2 seems to occur at higher ER [Ca^2+^], that is, at smaller decreases in ER, than the redistribution of STIM1 [Ca^2+^] [37]. In this perspective, STIM2 has been identified in an siRNA screen as a critical feedback regulator of basal cytosolic and ER [Ca^2+^] [37]. Knockdown of STIM2 markedly lowers the basal cytosolic and ER [Ca^2+^], while knockdown of STIM1 has less effect. However, these features may be dependent on the cell type and/or levels of STIM2, since basal [Ca^2+^] as well as the thapsigargin-releasable Ca^2+ ^did not differ between wild-type mouse embryonic fibroblast (MEF) cells and MEF cells deficient for STIM2 [38]. Similar findings were observed using an overexpression approach, in which STIM2 overexpression increased basal cytosolic [Ca^2+^], markedly more than STIM1 overexpression. Consistent with these findings, small reductions in ER [Ca^2+^] caused STIM2, but not STIM1, translocation and redistribution to punctae, with subsequent activation of Ca^2+ ^influx [37]. While both STIM1 and STIM2 have been implicated in triggering Ca^2+ ^influx following receptor-mediated ER Ca^2+^-store depletion [37,39], *STIM1*^*-/- *^cells displayed more severe defects in SOCE than *STIM2*^*-/- *^cells [38,39]. From agonist-induced Ca^2+ ^release in *STIM1*^*-/- *^and *STIM2*^*-/- *^cells, it was found that STIM2, but not STIM1, is dispensable for agonist-induced Ca^2+ ^signaling [38]. Nevertheless, the sustained nuclear localization of NFAT and production of cytokines are severely hampered in *STIM2*^*-/- *^T cells [39].

The fundamental biological role of STIM/Orai signaling is indicated by the fact that recessive mutations in STIM or Orai affecting their molecular function lead to severe hereditary immunodeficiency in humans [13]. Different mutations in STIM1 as well as Orai1 leading to loss-of-function or loss-of-expression of these proteins have been identified in immunodeficient patients [5,40-42]. Importantly, these defects can be overcome by STIM1 or Orai1 overexpression, but not by STIM2 or Orai2/3, indicating a predominant role for STIM1/Orai1 in T-cell Ca^2+^-signaling function. Loss of STIM1/Orai1 signaling in patients results in severe T-cell immunodeficiency and concomitant viral, bacterial and fungal infections [41-44]. Strikingly, a congenital, nonprogressive myopathy is consistently observed in infant patients, indicating that STIM1 not only plays a crucial role in T-cell activation and proliferation, but also in skeletal-muscle function and/or development (see part 3) [42].

### Other targets of STIM proteins

A detailed discussion on STIM targets can be found in [13].

#### Microtubule-plus-end-tracking protein EB1

STIM1 is recruited by end-binding protein-1 (EB1) to sites of physical contact between growing microtubule tips and ER [45,46]. However, the contribution of microtubule-associated STIM1 to Ca^2+ ^signaling is not clear. Preventing STIM1 localization at the microtubule does not directly affect SOCE [45] and microtubules are not essential for initial CRAC channel gating in T cells and mast cells [47,48]. Indirect effects of the association of STIM1 with the microtubule may be caused by remodeling of the ER or ER/plasma-membrane contact sites or the availability of STIM1.

#### Canonical transient receptor potential (TRPC) channels

Other candidates for SOCE include TRPC channels [25,49]. Biochemical and functional experiments revealed that STIM1 directly interacts with TRPC channels through electrostatic interaction, which involves the K864/K865 in the polybasic lysine-rich region of STIM1 and two negative charges that are conserved in all TRPC channels, that is, D639/D640 in TRPC1 and D697/D698 in TRPC3 [50]. STIM1 was shown to directly bind and regulate TRPC1, TRPC4 and TRPC5, while indirect actions of STIM1 on TRPC3 and TRPC6 have been proposed [49]. STIM1-dependent gating of TRPC channels seem to differ from their gating of Orai channels, pointing towards an independent gating mechanism of TRPCs versus Orai channels by STIM1 [50]. Nevertheless, the regulation of TRPC channels by STIM1 has been questioned in a very diligent study performed by the Putney lab [51], in which endogenous and ectopic TRPC1-, TRPC3-, TRPC5-, TRPC6- and TRPC7-mediated Ca^2+ ^entry was unaffected by increased or decreased STIM1 levels. A detailed discussion on the role of STIM1 in regulating Orai versus TRPC channels can be found in a recent review [52].

#### Arachidonate-regulated Ca^2+^-selective (ARC) channel

Another target of STIM1 is the ARC channel, a receptor-operated Ca^2+^-entry channel whose activation is completely independent of store depletion or of translocation of ER-resident STIM1 [53,54]. It is proposed that a fraction of STIM1 constitutively residing at the plasma membrane is responsible for the regulation of the activity of the ARC channels, since antibodies targeting plasmalemmal STIM1 or mutating its N-linked glycosylation sites essential for its cell-surface expression inhibited ARC-channel activity. Recent work identified the molecular architecture of ARC channels, revealing a pentameric organization consisting of three Orai1 and two Orai3 subunits [55]. This deviates from the tetrameric structure of Orai channels mediating CRAC currents.

#### Adenylate cyclase (AC)

A recent study revealed a novel role for STIM1 where it participated in the store-operated recruitment of AC [56]. Indeed, depleting ER Ca^2+ ^stores led to the recruitment of AC to the intracellular Ca^2+ ^stores, resulting in increased cAMP levels and enhanced signaling by protein kinase A. This process was shown to be independent of increases in cytosolic [Ca^2+^], but required the translocation of STIM1. This study therefore points out that STIM1 may be an important integrator molecule that mediates cross-talk between Ca^2+^-dependent signaling and cAMP-dependent signaling. Recently, STIM1-mediated store-operated cAMP signaling has been implicated in the downstream effects of Ca^2+ ^signaling induced by eicosapentaenoic acid, an omega-3 polyunsaturated fatty acid present in fish oil [57].

#### The L-type Ca^2+ ^channel Cav1.2

In addition to the well-described Orai targets of STIM1, two very recent studies identified the voltage-operated Ca^2+ ^channel Cav1.2 as a novel target of STIM1 [58,59]. STIM1 targeted the α1c pore-forming subunit via a direct interaction, thereby imposing an inhibitory control over Cav1.2 upon ER Ca^2+^-store depletion, independently of Orai1-channel activity or changes in cytosolic [Ca^2+^]. The mechanism involves the direct binding of CAD or SOAR to a region encompassing aa 1809-1908, located in the C-terminus of Cav1.2.

Importantly, while CAD or SOAR activates Orai1-mediated currents, they inhibit Cav1.2-mediated currents. The interaction of STIM1 with the C-terminus of Cav1.2 is critical for its inhibition. This process is independent of functional Orai1-channel activity, but Orai1 may help STIM1-mediated Cav1.2 inhibition by trapping STIM1 in punctae that contain Cav1.2 channels, thereby recruiting STIM1 in the vicinity of Cav1.2 channels. In addition, STIM1 expression seems to regulate the plasma-membrane level of Cav1.2 [59]. Overexpression of STIM1 caused a prominent decrease in the amount of Cav1.2 at the plasma membrane, leading to STIM1/Cav1.2 co-localization in intracellular vesicles and the internalization of the channels.

**Figure 1:**
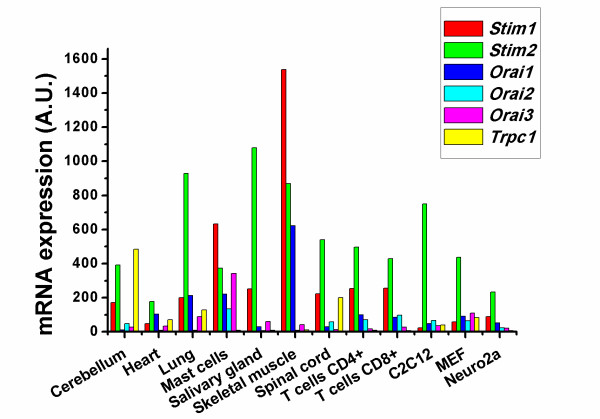
**Tissue distribution of Stim1, Stim2, Orai1-3 and Trpc1 in mouse**. Data were extracted from the BioGPS database (http://biogps.gnf.org/) and represent the mRNA expression patterns in selected mouse tissues [67,68]. *Stim1* is strongly expressed in skeletal muscle and mast cells, while *Stim2* exhibits a relatively higher expression level in all tissues tested with pronounced expression in salivary-gland tissue. *Orai1* expression is stronger in skeletal muscle, while there is low expression of all *Orai* in other tissues presented here. *Trpc1* expression is relatively low in both heart and skeletal-muscle tissue, but higher in tissue of the central nervous system, such as in the cerebellum. These patterns do not necessarily reflect the expression at the protein level or the situation in humans, but provide a good view of the patterns observed in mouse models.

The molecular targeting of STIM1 in Cav1.2 and Orai1-related Ca^2+^-influx pathways may be the molecular switch that accounts for the reciprocal regulation of voltage-gated Ca^2+ ^influx (inhibition) and store-operated Ca^2+ ^influx (activation) [58,59]. Interestingly, only excitable cells are able to increase cytosolic [Ca^2+^] in response to membrane depolarization, although immune cells (T cells, B cells, dendritic cells and mast cells) also express voltage-gated Ca^2+ ^channels [60,61]. In contrast, only non-excitable cells display prominent CRAC-channel activity upon store depletion, while SOCE is only a minor component of Ca^2+ ^influx in excitable cells [62,63]. However, excitable cells, like smooth-muscle cells [64,65], neurons [66] and skeletal-muscle cells [63], do express STIM1. The high expression level of STIM1 in skeletal muscle is supported by data obtained from BioGPS (http://biogps.gnf.org/), an online gene annotation portal (Figure 1) [67,68]. Since Cav1.2 and Orai activate different downstream Ca^2+^-signaling cascades that control growth [65], differentiation [63] and cell death [66], it is likely that STIM1 is a novel key player with different functions in these cell types.

### STIM1 in skeletal muscle

#### SOCE mechanism in skeletal muscle

SOCE in skeletal muscle was originally described in a study from Kurebayashi and Ogawa [69]. They discovered in thin muscle-fiber bundles of the extensor digitorum longus (EDL) muscle of adult mice, that the depletion of the SR by repetitive high-K^+ ^stimulation in the presence of sarcoplasmic/endoplasmic-reticulum Ca^2+ ^ATPase (SERCA) inhibitors triggered SOCE with the same characteristics as the CRAC current. This pathway is distinct from the excitation-coupled Ca^2+ ^entry, which is store-independent [62]. Both pathways consist of distinct molecular components and activation mechanisms [62]. Ca^2+ ^entry is important for store repletion [69], limiting fatigue under conditions of extensive exercise [70], activation of NFAT [63,71] and muscle differentiation [72]. Hence, it is becoming increasingly clear that dysregulation of Ca^2+ ^entry may lead to severe muscle pathologies [73-75]. In general, we will limit our discussion to SOCE and we will refer to other reviews for excitation-coupled Ca^2+ ^entry [76,77].

**Figure 2:**
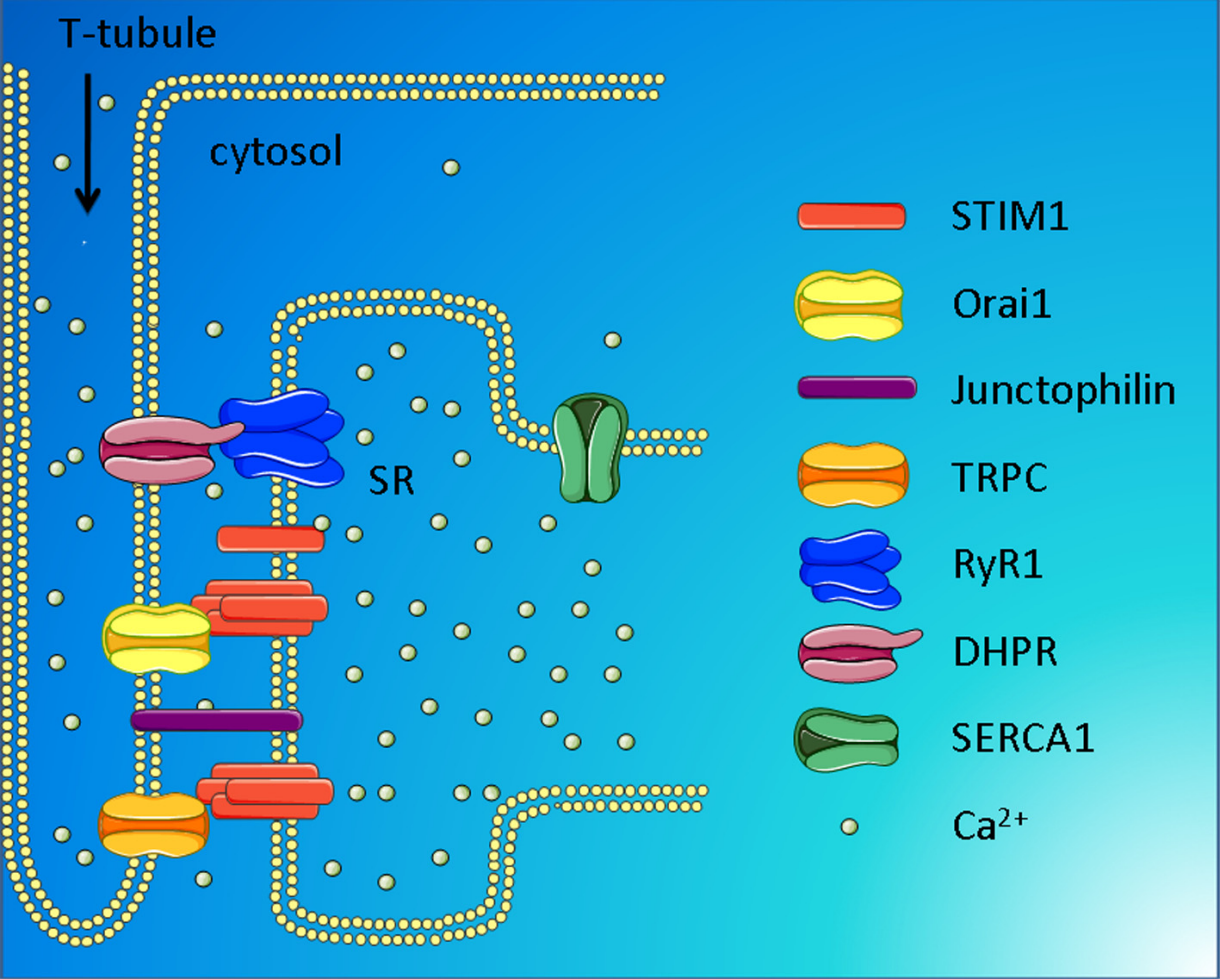
**Regulators of SOCE in skeletal muscle**. At the triad junction, the voltage-sensitive DHPR (Cav1.1) and RyR physically interact. Depolarization of the plasma membrane causes activation of DHPR and subsequent opening of intracellular Ca^2+^-release channels, like the most abundant RyR isoform in skeletal muscle, RyR1. In addition, STIM1 monomers and/or oligomers accumulate at the terminal cisternae of the SR, thereby being in close proximity or in complex with Orai1. This pre-localization of STIM1 with Orai1 likely accounts for the fast-activation kinetics of SOCE. Furthermore, it may also account for a basal Ca^2+ ^influx through Orai1 channels in resting conditions. As a consequence, STIM1/Orai1 complexes are an integral part of the Ca^2+^-homeostasis mechanisms responsible for maintaining proper SERCA1-mediated filling of the SR Ca^2+ ^stores and sustaining resting [Ca^2+^] in the cytosol. The highly specialized and structural organization of the triad in close proximity of the SR to plasma-membrane SOCE, which involves junctophilin, seems to be essential for proper STIM1/Orai1-mediated Ca^2+ ^homeostasis. A similar mechanism involving TRPC channels and STIM1 oligomers have also been implicated in Ca^2+^-influx mechanisms in the skeletal muscle, although STIM1-dependent regulation of TRPCs is a matter of debate. Abbreviations: DHPR, dihydropyridine receptor; RyR, ryanodine receptor; SOCE, store-operated Ca^2+ ^entry; STIM, stromal interaction molecule; SR, sacroplasmic reticulum; TRPC, canonical transient receptor potential.

Four models for SOCE in skeletal muscle have been proposed, including the conformation coupling between i) ryanodine receptors (RyRs) and TRPCs, ii) inositol 1,4,5-trisphosphate receptors (IP_3_Rs) and TRPCs, iii) STIM1 and Orai1 and iv) STIM1 and TRPC channels [76]. Figure 2 shows the molecular determinants involved in SOCE in skeletal muscle with indication of the outside-inside coupling between the dihydropyridine receptor (DHPR), the skeletal-muscle type-1 RyR (RyR1) and the inside-outside SOCE-coupling mechanisms via STIM1/Orai1 and STIM1/TRPC.

Different studies proposed a role for conformational coupling of RyRs to TRPCs, thereby activating SOCE through TRPC channels [71,73,78]. However, RyRs are likely not essential for SOCE in skeletal muscle, since myotubes of mice lacking RyR1/RyR3 still display prominent SOCE [62,70]. Consistent with this, Lee and *et al. *did not find any role for TRPC3 in Ca^2+ ^entry in skeletal muscle although its expression level increased during differentiation [79]. The authors proposed that the functional interaction between RyR1 and TRPC3 enhances RyR1 Ca^2+^-release-channel activity and is thus required for adequate SR Ca^2+ ^release.

Another candidate proposed was the coupling between IP_3_Rs and TRP-family members [80,81], like TRPC3 [82]. However, the expression level of IP_3_Rs in myotubes is relatively low and their localization is rather around the nuclear envelope than at the SR terminal cisternae (TC) [83,84].

The identification of STIM1 as the ER Ca^2+^-sensor protein and its conformational coupling to Orai1 channels controlling SOCE in T lymphocytes spurred the idea that STIM1/Orai1 may be the molecular players underlying SOCE also in skeletal muscle. Different lines of evidence support the idea that STIM1 is critical for SOCE in skeletal muscle: i) STIM1 and Orai1 are highly expressed in skeletal muscle (Figure 1) [63,85], ii) STIM1 is pre-localized at the SR junctions with the T-tubule system which contains pre-localized Orai1 [63,85], iii) mice lacking STIM1 or Orai1 display myopathy [63], iv) severe combined immunodeficiency (SCID) patients characterized by loss-of-function mutations in STIM1/Orai1 signaling display skeletal-muscle myopathy [5], and v) knockdown of STIM1 or expression of the dominant negative Orai1 E106Q caused a marked decline in SOCE in skeletal-muscle myotubes [62].

Besides Orai channels, a role for a conformational coupling between STIM1 and TRPC channels in skeletal muscle can however not be excluded. TRPC1 and TRPC3 channels have been shown to be expressed in skeletal muscle and have been implicated in SOCE in lymphocytes [25]. Moreover, STIM1/Orai1/TRPC1-ternary complexes have been shown to assemble during store depletion, thereby contributing to SOCE [49,50,86]. The C-terminal domain of STIM1 has been shown to directly bind and activate TRPC1 upon ER Ca^2+^-store depletion. Furthermore, in a recent study using whole-cell patch-clamp recordings approximately 60% of primary myotubes displayed an inwardly rectifying current with characteristics typical for CRAC current, while approximately 40% of myotubes displayed linear current-voltage relationships, which may be related to store-operated activation of TRPC channels [63]. These observations are in line with evidence obtained from myoblasts, which displayed a decrease in SOCE upon repression of TRPC1 expression, thereby affecting myoblast migration and differentiation [87]. However, while mice lacking TRPC1 display a muscle phenotype with muscle fibers that have a decreased cross-sectional area, a reduced force generation, a decline in the level of myofibrillar proteins and a decreased resistance towards muscle fatigue, the role of TRPC1 in adult fibers seemed independent of the Ca^2+^-store content [88]. The work of Gailly and others indicates that the role of TRPC1 in SOCE is dependent on factors that differ among myoblasts and adult fibers [87,88]. A pivotal role for the a-isoform of the inhibitor of myogenic family (I-mfa) has been proposed, since I-mfa binds and inhibits TRPC1 and myogenic factors interfere with these complexes to alleviate TRPC1 inhibition by I-mfa [89,90]. Hence, in myoblasts, TRPC1 may drive the onset of differentiation through a store-controlled mechanism, while in adult fibers TRPC1 may sustain endurance by maintaining force production upon repeated stimulation and proper muscle development through a store-independent mechanism.

#### STIM1/Orai1 pre-localization in skeletal muscle and consequences for SOCE activation

While the molecular determinants responsible for SOCE are very similar among T lymphocytes and skeletal-muscle cells, there are also some striking differences, which may be related to the pre-localization of STIM1/Orai1 and the putative contribution of voltage-gated Ca^2+ ^channels in the targeting of STIM1 to the TC/T-tubule junctions. Indeed, a clear distinction between SOCE in T lymphocytes and in skeletal muscle is the kinetics of CRAC-channel activation. In T lymphocytes, the kinetics of SOCE activation by store depletion is relatively slow with a delay of tens of seconds between store depletion and SOCE. In contrast, in skeletal muscle the local activation of SOCE by store depletion is almost instant (less than 1 second delay) and graded in nature, as shown in recent studies by Launikonis [91,92]. Hence, it was proposed that pre-localization of STIM1 and Orai1 at TC/T-tubule junctions under basal conditions when SR stores are fully loaded with Ca^2+ ^underpins the fast kinetics (Figure 2). The pre-localization of STIM1 in the SR at triad junctions was observed in differentiated myotubes of the C2C12 cell line as well as in native skeletal muscle from the hind limbs of adult mice [63,85]. The exact molecular architecture of the inactive STIM1/Orai1 complexes in resting skeletal-muscle cells remains to be elucidated. Two models that allow for rapid SOCE activation upon SR Ca^2+^-store depletion were proposed by Dirksen [76]. In model 1, STIM1 monomers are localized in the vicinity of inactive Orai1 channels at the triad junctions. A decrease in the SR [Ca^2+^] will cause rapid dissociation of Ca^2+ ^from STIM1, resulting in conformational changes in STIM1, its oligomerization and activation of the pre-localized Orai1 channels. In model 2, STIM1 exists in pre-formed complexes with the C-terminal region of inactive Orai1 channels, which remain silent until a decrease in SR [Ca^2+^] triggers conformational changes in STIM1 and direct activation of Orai1-mediated Ca^2+ ^influx, for example through an interaction of STIM1 with the N-terminus of Orai1. The latter would allow for an ultra-fast, efficient and tightly controlled activation of Orai1.

In any case, the spatial organization of triad-junction structure, which allows close contacts between the TC of the SR and the transverse tubular invaginations of the plasma membrane, seems to be the key for efficient SOCE in skeletal muscle [93]. Indeed, disrupting the triad structure in skeletal-muscle fibers by acute suppression of junctophilin 1/2, a protein responsible for forming the close contacts between the intracellular stores and the plasma membrane (Figure 2), leads to reduced SOCE, a decrease in the SR Ca^2+ ^content and altered caffeine-triggered RyR-mediated Ca^2+ ^response. Hence, structural properties of the triad junctions seem to be responsible for the efficient coupling of retrograde signaling from the SR to T-tubules, thereby controlling SOCE, and overall Ca^2+ ^homeostasis and muscle physiology. These features may be important in explaining the effect of pathophysiological mutations in junctophilins or altered junctophilin-expression profiles that are associated with cardiac failure or muscle aging [94,95]. Very recently, reduced SOCE in junctophilin-1 knock out myotubes was associated with a decline in STIM1/Orai1-expression levels and a reduced resting cytosolic [Ca^2+^] and SR Ca^2+ ^content [96]. Using a Ca^2+^-entry blocker, BTP2, Eltit and co-workers showed that Ca^2+ ^influx was essential for maintaining proper resting [Ca^2+^], since treating wild-type myotubes with BTP2 caused a decrease in SOCE and in resting [Ca^2+^], resembling the situation in junctophilin-1 knock out myotubes [96]. Since different Ca^2+^-entry mechanisms may account for this pharmacological effect, the authors elegantly used the dominant negative Orai1 form, Orai1 E190Q. Strikingly, expression of Orai1 E190Q was sufficient to inhibit SOCE in wild-type myotubes and to decrease the resting [Ca^2+^], while it had no effect in junctophilin-1 knock out myotubes. As a consequence, wild-type myotubes expressing Orai1 E190Q displayed a reduced SR Ca^2+ ^content. Hence, this study is one of the first to show that Orai1-mediated SOCE is critical to control resting cytosolic [Ca^2+^] and SR Ca^2+ ^content. This further supports the concept that SOCE is an essential feature for proper muscle function, not only during conditions of intensive stimulation, but also during resting conditions.

It is also conceivable that other plasma-membrane channels may contribute to the pre-localization of STIM1 to the TC/t-tubule junctions. The identification of the α1c subunit of the voltage-gated Cav1.2 channel as a novel target of STIM1 [58,59] may point towards a more general role for voltage-gated Ca^2+ ^channels to target STIM1 in spatially or functionally restricted domains. Although the DHPR L-type Ca^2+^-channel (α1s) differs in properties with α1c, it is possible that STIM1 interacts with both.

#### STIM1 in physiological signaling

**Figure 3:**
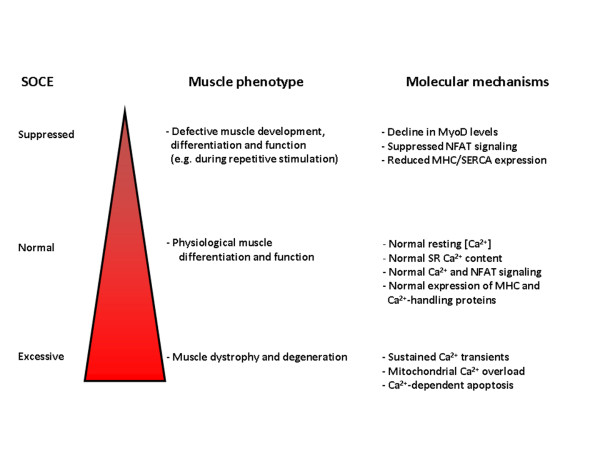
**Schematic overview of the physiological role of SOCE in skeletal muscle and pathophysiological consequences of Ca**^**2+**^**-influx dysregulation based on mouse-model studies**. STIM1-controlled Ca^2+ ^influx, either through Orai1 or TRPC channels, ought to be tightly regulated for proper muscle development and function. STIM1-gated Orai1-mediated Ca^2+ ^influx seems to be a requisite for proper Ca^2+ ^homeostasis in the skeletal muscle, maintaining resting cytosolic [Ca^2+^] sufficiently high and adequately filling the SR Ca^2+ ^stores. This constitutes an essential mechanism that compensates for Ca^2+ ^losses to the extracellular space. Importantly, these phenomena may not only act during periods of intense stimulation, but may also be required during basal conditions and regular muscle function. On the one hand, suppressing Ca^2+ ^influx, for example in STIM1-deficient muscle fibers, leads to improper muscle development and function. This is likely due to hampered activation of NFAT-dependent signaling and defective expression of Ca^2+^-handling proteins, such as SERCAs and RyRs, as well as other proteins involved in muscle contraction. On the other hand, events such as mutations in dystrophin or overexpression of TRPC channels, lead to muscle dystrophy and degeneration, likely due to mitochondrial Ca^2+ ^overload and Ca^2+^-dependent activation of the apoptotic program. Abbreviations: NFAT, nuclear factor of activated T cells; RyR, ryanodine receptor; SERCA, sarcoplasmic/endoplasmic-reticulum Ca^2+^-ATPase; SR, sacroplasmic reticulum; STIM, stromal interaction molecule; TRPC, canonical transient receptor potential.

Recently, it became clear that SOCE plays a prominent role in muscle development and muscle function and that STIM1 hereby has a central role [63,93,96,97] (Figure 3). For example, mice deficient in STIM1 signaling displayed defects in muscle differentiation and contractile activity [63]. Homozygous STIM1-deficient neonatal mice died from a perinatal myopathy, whereas STIM1-haploinsufficient mice displayed increased susceptibility to fatigue. These data indicate that STIM1 controls both chronic Ca^2+^-controlled signaling processes, like muscle differentiation and remodeling through the activation of a genetic program, as well as acute Ca^2+^-signaling processes such as muscle contraction, by supporting the adequate function of the contractile system under conditions of prolonged motor-nerve activity.

Underlying these phenomena is STIM1-dependent activation of NFAT in skeletal muscle, which drives myogenesis and muscle differentiation [63]. It has been known for a long time that a dramatic remodeling of Ca^2+^-transport mechanisms underlies and accompanies skeletal-muscle differentiation. Differentiation of BC_3_H1 cells to a muscular phenotype was characterized by a decrease in IP_3_-induced Ca^2+ ^release and an increase in Ca^2+^-pump activity as well as in caffeine-induced Ca^2+ ^release [98]. Also during C2C12 differentiation from myoblasts to multinucleated myotubes, IP_3_R-expression levels declined, while the expression levels of RyR1 Ca^2+^-release channels and SERCA2a/SERCA1 Ca^2+ ^pumps dramatically augmented [99]. A recent study of Stiber *et al. *[63] now adds STIM1 as another molecular factor, whose expression level increases and whose localization changes from perinuclear to cell peripheral upon differentiation of C2C12 myoblasts into myotubes. These molecular findings correlate with the increased rate of Ca^2+ ^influx in these cells upon thapsigargin-induced SR Ca^2+^-store depletion.

**Figure 4:**
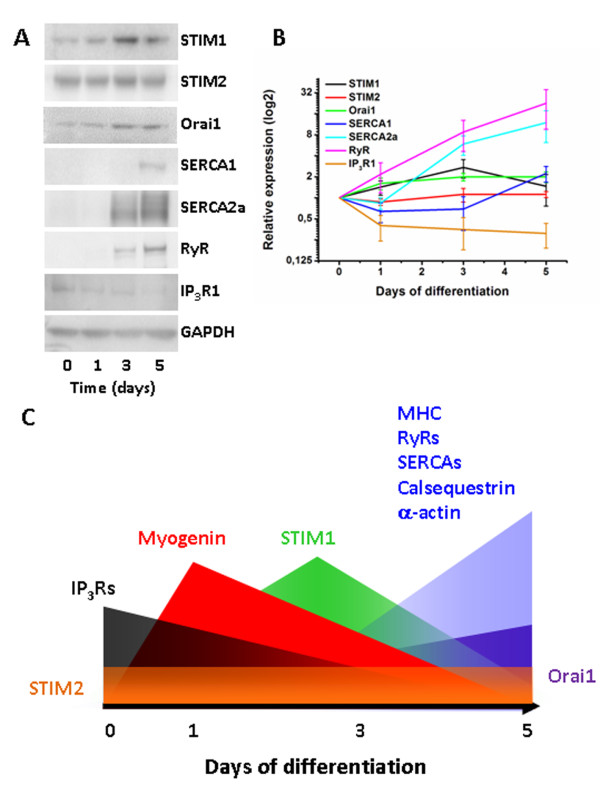
**Expression of Ca**^**2+**^**-handling proteins including STIM1, STIM2 and Orai1 during myogenic differentiation of C2C12 cells**. A: Representative panel of Western blots for each protein of interest in lysates from C2C12 cells in an undifferentiated state and different stages of differentiation. B: Graph represents the average expression of three independent biological samples (mean ± S.E.M.) of C2C12 cells undergoing differentiation. All values were normalized to GAPDH, which was used as a loading control. C: A model for key proteins involved in the proper remodeling of myoblasts into myotubes, based on previous reports [99] and own data. The expression pattern of myogenin, taken from the study of MacLennan and co-workers [99], was up-regulated starting from day 1 of myoblast differentiation, which would mark the first wave of differentiation [99]. Orai1, SERCA2a and RyR1 started to emerge at day 3, while cells exhibit a transient cardiac phenotype. Interestingly, we also observed an even shorter transient of STIM1 up-regulation, which seemed to decline at the same time as SERCA1 levels increased. Remarkably, while STIM1 was transiently up-regulated, STIM2 did not significantly alter and Orai1 continued to increase during differentiation. Materials and methods: Cells were collected 0, 1, 3 or 5 days after replacing myoblast culture medium with myotube differentiation medium [99,113]. Cell lysates were analyzed for protein expression by Western blot and expression was each time normalized to GAPDH expression. Signals were detected with ECF and analyzed with ImageJ. Antibodies were from ProSci Inc., Poway, CA, USA (Orai1), Sigma, St Louis, MO, USA (STIM2 and GAPDH), Abnova, Taipei City, Taiwan (STIM1) and Thermo Scientific, Rockford, IL, USA (SERCA1 and RyR). The SERCA2a and IP_3_R1 antibodies are described elsewhere [114,115]. Abbreviations: ECF, enhanced chemifluorescence; GAPDH, glyceraldehyde 3-phosphate dehydrogenase; IP_3_R, inositol 1,4,5-trisphosphate receptor; RyR, ryanodine receptor; SERCA, sarcoplasmic/endoplasmic-reticulum Ca^2+^-ATPase; STIM, stromal interaction molecule.

Independently, we have examined STIM1- and STIM2-protein levels together with the expression of other Ca^2+^-handling proteins, like Orai1, SERCA1, SERCA2a, RyRs and IP_3_Rs in differentiating C2C12 cells (Figures 4A and 4B). We could confirm the up-regulation of STIM1 and Orai1 in C2C12 cells undergoing differentiation. However we observed a transient up-regulation of STIM1, while Orai1 is permanently augmented. Strikingly, the maximal STIM1 levels correlate with the up-regulation of Orai1, but precede the up-regulation of SERCA1, SERCA2a and RyR. In contrast, STIM2 levels did not significantly change during C2C12 differentiation, suggesting a selective role for STIM1 in skeletal-muscle differentiation. STIM1 up-regulation seems to be a very proximal event in skeletal-muscle differentiation. Nevertheless, this transient STIM1 up-regulation seems to correlate with the observations of Stiber *et al. *in muscle formation during development *in vivo *[63].

It is possible that the transient STIM1 up-regulation, which seems to occur as an intermediate step between myogenin and SERCA/RyR up-regulation, is needed for increasing Ca^2+ ^influx and is required for driving the remodeling of the Ca^2+^-handling proteins through Ca^2+^-dependent NFAT signaling (Figure 4C). Indeed, Stiber *et al. *have shown that STIM1 silencing with short hairpin RNA decreased basal NFAT trans-activation in differentiated myotubes, while constitutively active STIM1 expression increased basal NFAT trans-activation [63]. Furthermore, NFAT is known to control muscle formation through morphogenetic events that depend on Ca^2+^-dependent signaling through the activation of calcineurin/NFAT [100,101].

The critical role of STIM1 in skeletal-muscle development was further demonstrated by Stiber *et al. *using a gene trap approach resulting in the expression of STIM1/LacZ-fusion proteins (STIM1^gt^), which contained the N-terminal EF-SAM domain and the transmembrane domain of STIM1 [63]. Most homozygous *STIM1*^*gt/gt *^mice died at a neonatal stage. However, surviving mice displayed decreased body weight, impaired muscle formation and increased fatigue. Studies revealed that the SOC currents in response to thapsigargin were completely abolished in primary myotubes deficient of STIM1.

STIM1 deficiency caused a severe impairment of the skeletal-muscle structure and function. STIM1 loss also resulted in increased central nucleation, a reduced muscle cross-sectional area, swollen mitochondria and a decline in the muscle-specific proteins in the SR, like SERCA1 and myosin heavy chain. In addition, the levels of MyoD, one of the master regulatory genes that controls muscle differentiation, were lower in the muscle of STIM1-deficient mice. At the functional level, loss of STIM1 led to a decrease in the maximal tetanic force and in the fatigue resistance. The latter could be attributed to an impaired SR Ca^2+^-store filling in the myotubes. After on-going depolarization pulses, the content of the SR Ca^2+ ^stores was severely reduced in *STIM1*^*gt/gt *^myotubes in contrast to their wild-type counterparts. This indicates that STIM1-controlled SOCE is required to refill the internal SR Ca^2+ ^stores during repeated stimulations. The latter has been confirmed by another study showing the rapid efflux of Ca^2+ ^in the T-tubule system upon muscle-cell depolarizations [91]. A critical function of STIM1 in SR Ca^2+^-store filling in human skeletal muscle needs to be confirmed. However, the fact that SCID patients carrying loss-of-protein expression mutations in the *STIM1 *gene, manifest atrophy of the type-II skeletal-muscle fibers resulting in severe chronic pulmonary disease [41], supports the concept that STIM1-mediated SOCE plays a critical role in sustaining proper SR Ca^2+^-store filling and skeletal-muscle function in humans.

#### STIM1 in pathophysiological signaling

While STIM1 and SOCE seem to be essential for skeletal-muscle development, excessive store-operated Ca^2+ ^influx may underpin pathological conditions, like Duchenne muscular dystrophy [74]. A crucial feature of this disease is defective expression of dystrophin. Myotubes lacking functional dystrophin display altered Ca^2+ ^dynamics, characterized by exaggerated SOCE [74,102]. The latter may be responsible for the observed sustained cytosolic Ca^2+ ^transients and increased Ca^2+ ^uptake by the mitochondria. Importantly, re-introduction of minidystrophin reduces Ca^2+ ^entry to its normal level, which leads to shorter Ca^2+ ^transients and decreased mitochondrial Ca^2+ ^uptake [74]. Although these studies did not clarify the involvement or role of STIM1, they point towards a critical regulation of proper SOCE for the physiological function of skeletal cells. Indeed, SOCE apparently needs to be tightly regulated, since suppressed as well as exaggerated SOCE underpin skeletal-muscle dysfunction [63,74]. An elegant study recently published by the Molkentin lab indicated that increased Ca^2+ ^entry by itself is sufficient to induce muscular dystrophy *in vivo*, since transgenic mice overexpressing TRPC3 channels are characterized by features similar to the dystrophic disease models [75].

Different molecular mechanisms of increased Ca^2+ ^entry underpinning this disease model have been proposed, including store-operated and stretch-operated ion channels [103]. On the one hand, TRPC channels seem important candidates [75]. Indeed, mdx dystrophic skeletal-muscle fibers displayed increased TRPC-mediated Ca^2+ ^influx [73,75]. Interestingly, TRPC1 has been shown to associate with the dystrophin-protein complex [104,105]. Moreover, dystrophic skeletal-muscle disease models associated with mutations in the dystrophin or mutations in the delta-Sarcoglycan (*Scgd*) genes were rescued by transgene-mediated inhibition of TRPC channels, thereby reducing Ca^2+ ^influx and preventing the development of muscular-dystrophy features [75]. On the other hand, Launikonis and co-workers demonstrated that while SOCE functions normally in mdx muscle fibers, the thresholds for activation and deactivation of SOCE have been shifted to higher SR [Ca^2+^] [106]. This may contribute to higher Ca^2+ ^influx during long periods of stimulation. The molecular basis for these shifts in threshold may be associated with the dramatic increase in STIM1/Orai-protein levels found in the mdx muscle fibers [106]. Interestingly, these data may correlate with our observations suggesting a transient up-regulation of STIM1 during muscle differentiation, while a permanent up-regulation of STIM1 may contribute to deleterious muscle events.

Independently of the mechanism, preventing Ca^2+ ^influx may be beneficial and holds potential for future therapeutic strategies to tackle muscular dystrophy either by targeting STIM1/Orai1 or TRPC channels, since both channel complexes have been shown to be implicated and/or up-regulated in Duchenne muscular dystrophy models [75,106]. All studies seem to agree on the fact that excessive SOCE is an early event in this pathology and that inhibiting SOCE seems to be beneficial. This paradigm is supported by different studies: i) TRPC suppression rescues muscular dystrophic features in mouse models [75]; ii) blockers of stretch-activated channels prevent muscle degeneration in mdx mice [107]; iii) inhibitors of phospholipase A2, which is overexpressed in skeletal muscle of a mouse model for Duchenne muscular dystrophy, attenuate the exaggerated SOCE and the subsequent muscle damage [102].

Furthermore, while reducing Ca^2+ ^influx may be critical for targeting this pathology, inhibiting Ca^2+ ^release from the ER has also been shown to be beneficial [108]. For instance, overexpression of anti-apoptotic Bcl-2 prevented IP_3_R-mediated Ca^2+ ^release [109,110] and subsequent mitochondrial Ca^2+ ^overload, thereby protecting dystrophic muscle cells against Ca^2+^-dependent apoptosis [108]. Hence, a concerted strategy to alleviate muscular dystrophy likely requires the dampening of both Ca^2+ ^influx as well as ER Ca^2+ ^release.

**Figure 5:**
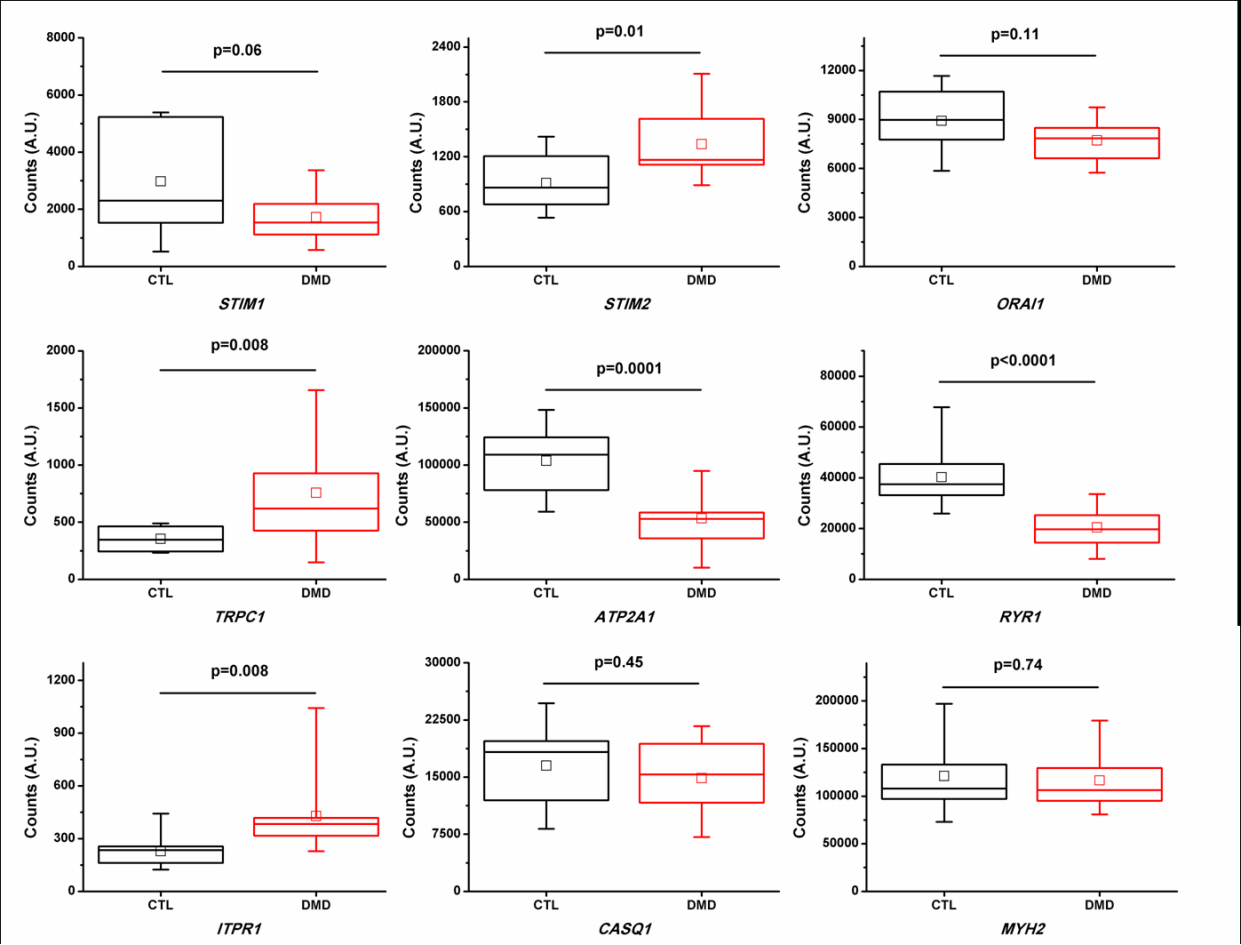
**Gene expression involved in Ca**^**2+ **^**and/or contractility**. Plots represent gene expression in quadriceps skeletal-muscle samples of controls (CTL, n = 10-12) and patients with Duchenne muscular dystrophy (DMD, n = 10 - 12) in arbitrary units (A.U.). Data were obtained from GEO reference series GSE1007 (*STIM1*, *STIM2*, *ORAI1*, *ITPR1 *and *CASQ1*) and GSE1004 (*TRPC1*, *ATP2A1*, *RYR1 *and *MYH2*) [116-118] comparing the mRNA-expression levels in normal and dystrophic patients (http://www.ncbi.nlm.nih.gov/geo/). Graphs represent box plots, indicating the mean (square symbol), the median (line), the 25^th ^and 75^th ^percentiles (bottom and top of the box), and the 5^th ^and 95^th ^percentiles (whisker range). Strikingly, STIM1-, SERCA1-, RyR1-mRNA levels tended to decline, while STIM2-, TRPC1- and IP_3_R1-mRNA levels tended to increase. Orai1-, calsequestrin-1- and myosin heavy chain-2-mRNA levels did not significantly alter. This seems opposite to what has been observed in mouse models for Duchenne muscular dystrophy, which displayed excessive Ca^2+ ^influx and up-regulation of STIM1/Orai1 [106]. In human patients suffering from Duchenne muscular dystrophy, TRPC1 elevations may account for the increase in Ca^2+ ^influx, leading to Ca^2+^-dependent apoptosis and muscle degeneration. This indicates that caution should be taken from extrapolating results from mouse models for pathophysiological conditions to human pathophysiological conditions. Abbreviations indicate the gene names for stromal interaction molecule 1 (*STIM1*), stromal interaction molecule 2 (*STIM2*), Orai1 (*ORAI1*), canonical transient receptor potential 1 (*TRPC1*), sarcoplasmic/endoplasmic-reticulum Ca^2+^-ATPase 1 (*ATP2A1*), ryanodine receptor 1 (*RYR1*), inositol 1,4,5-trisphosphate receptor 1 (*ITPR1*), calsequestrin 1 (*CASQ1*), and myosin heavy chain IIa (*MYH2*).

Finally, it is important to note that most of the evidence that points towards excessive Ca^2+ ^influx as an early event in the development of muscular dystrophy has been obtained from mouse models. We need to keep in mind that the need and physiological role of SOCE might be different for mouse and human skeletal muscle. For instance, a micro-array analysis of human skeletal-muscle biopsies from control patients and Duchenne muscular dystrophy patients (obtained from the Gene Expression Omnibus; http://www.ncbi.nlm.nih.gov/geo/) indicated that the STIM1-mRNA levels were not increased, but rather tended to decrease in the muscle of the dystrophic patients, while Orai1-mRNA levels were not significantly changed (Figure 5). This seems in contrast with the up-regulation of STIM1 and Orai1 and the increased SOCE reported in mouse models for Duchenne muscular dystrophy. These contrasting findings may indicate that mechanisms underpinning SOCE are differently affected in the human muscular pathologies versus the mouse models for these pathologies. In addition, the relative importance of STIM1 versus STIM2 for SOCE in human muscles and their contribution to myopathies may differ among human and mice. In this respect, Duchenne muscular dystrophy patients did show an up-regulation of STIM2-mRNA levels, which is activated at more modest decreases in [Ca^2+^]_ER _than STIM1. Therefore, STIM2 up-regulation may be another critical factor that needs to be taken into account in the development of muscular dystrophy in human patients. In any case, it is clear that the role of both STIM proteins for the development of myopathies in human patients must be further explored.

Moreover, other mechanisms may account for the excessive Ca^2+ ^influx that leads to muscle degeneration in human patients. Therefore, it is important to note that TRPC1-mRNA levels are also significantly up-regulated in the patients suffering from muscular dystrophy. Importantly, excessive TRPC1 activity has been implicated in spontaneous Ca^2+ ^influx and the activation of Ca^2+^-dependent proteolysis, leading to the degradation of cytoskeletal proteins and the development of myopathies in Homer 1-deficient mice [111]. Decreased levels of Homer 1 in mdx mouse models may contribute to the reported TRPC1 hyperactivity in response to store depletion [73]. However, the contribution of STIM1 in this process remains unknown.

Finally, these micro-array analyses also revealed that SERCA1- and RyR1-mRNA levels declined, IP_3_R1-mRNA levels increased and Orai1-, calsequestrin-1- and myosin heavy chain-2-mRNA levels did not significantly alter. Changes in IP_3_R-expression level have previously been shown to occur in patient samples of Duchenne muscular dystrophy [112]. This indicates a remodeling of the Ca^2+^-handling mechanisms that tends to shift to the undifferentiated state, while proteins involved in the contractile mechanism are not profoundly affected. In any case, it would be interesting to elucidate whether the changes in Ca^2+^-transport mechanisms correlate with the severity of the disease.

## Conclusions

While the role of SOCE is well established in T cells and other non-excitable cells, it is becoming clear that SOCE may play a very important role in skeletal-muscle physiology, ranging from roles in development, differentiation, contractile function and resistance against fatigue. STIM1 plays a central role in these processes by controlling SOCE channels, like Orai1 and/or TRPCs in a rapid and highly regulated fashion. While physiological SOCE is required for proper muscle development and function, excessive SOCE seems to be detrimental for skeletal muscle. Indeed, promoting Ca^2+ ^influx by overexpressing TRPCs is sufficient to degenerate healthy muscle, while genetic mouse models for muscular dystrophy have been characterized by excessive SOCE. From these studies, it is clear that STIM1 function and thus SOCE ought to be tightly regulated for proper muscle physiology.

## List of Abbreviations

CRAC: Ca^2+^-release-activated Ca^2+^; DMD: Duchenne muscular dystrophy; ER: endoplasmic reticulum; GAPDH: glyceraldehyde 3-phosphate dehydrogenase; IP_3_R: inositol 1,4,5-trisphosphate receptor; NFAT: nuclear factor of activated T cells; RyR: ryanodine receptor; SCID: severe combined immunodeficiency; SERCA: sarcoplasmic/endoplasmic-reticulum Ca^2+^-ATPase; SOCE: store-operated Ca^2+ ^entry; SPCA: secretory-pathway Ca^2+ ^ATPase; SR: sarcoplasmic reticulum; STIM: stromal interaction molecule; TC: terminal cisterna(e); TRPC: canonical transient receptor potential.

## Competing interests

The authors declare that they have no competing interests.

## Authors' contributions

GB conceived the experiments, analyzed the data and wrote the manuscript. SK performed the experiments, analyzed the data and wrote the manuscript. JPD, HDS, LM and JBP discussed the data and revised the manuscript.

## References

[B1] Putney JW Jr A model for receptor-regulated calcium entry Cell Calcium 1986 7 1 12 10.1016/0143-4160(86)90026-6 2420465

[B2] Takemura H Hughes AR Thastrup O Putney JW Jr Activation of calcium entry by the tumor promoter thapsigargin in parotid acinar cells. Evidence that an intracellular calcium pool and not an inositol phosphate regulates calcium fluxes at the plasma membrane J Biol Chem 1989 264 12266 12271 2663854

[B3] Roos J DiGregorio PJ Yeromin AV Ohlsen K Lioudyno M Zhang S Safrina O Kozak JA Wagner SL Cahalan MD Veliçelebi G Stauderman KA STIM1, an essential and conserved component of store-operated Ca^2+ ^channel function J Cell Biol 2005 169 435 445 10.1083/jcb.200502019 15866891 PMC2171946

[B4] Liou J Kim ML Heo WD Jones JT Myers JW Ferrell JE Jr Meyer T STIM is a Ca^2+ ^sensor essential for Ca^2+^-store-depletion-triggered Ca^2+ ^influx Curr Biol 2005 15 1235 1241 10.1016/j.cub.2005.05.055 16005298 PMC3186072

[B5] Feske S Gwack Y Prakriya M Srikanth S Puppel SH Tanasa B Hogan PG Lewis RS Daly M Rao A A mutation in Orai1 causes immune deficiency by abrogating CRAC channel function Nature 2006 441 179 185 10.1038/nature04702 16582901

[B6] Vig M Peinelt C Beck A Koomoa DL Rabah D Koblan-Huberson M Kraft S Turner H Fleig A Penner R Kinet JP CRACM1 is a plasma membrane protein essential for store-operated Ca^2+ ^entry Science 2006 312 1220 1223 10.1126/science.1127883 16645049 PMC5685805

[B7] Zhang SL Yeromin AV Zhang XH Yu Y Safrina O Penna A Roos J Stauderman KA Cahalan MD Genome-wide RNAi screen of Ca^2+ ^influx identifies genes that regulate Ca^2+ ^release-activated Ca^2+ ^channel activity Proc Natl Acad Sci USA 2006 103 9357 9362 10.1073/pnas.0603161103 16751269 PMC1482614

[B8] Prakriya M Feske S Gwack Y Srikanth S Rao A Hogan PG Orai1 is an essential pore subunit of the CRAC channel Nature 2006 443 230 233 10.1038/nature05122 16921383

[B9] Smyth JT Hwang SY Tomita T DeHaven WI Mercer JC Putney JW Activation and regulation of store-operated calcium entry J Cell Mol Med 2010 14 2337 2349 10.1111/j.1582-4934.2010.01168.x 20807283 PMC3074973

[B10] Peinelt C Vig M Koomoa DL Beck A Nadler MJ Koblan-Huberson M Lis A Fleig A Penner R Kinet JP Amplification of CRAC current by STIM1 and CRACM1 (Orai1) Nat Cell Biol 2006 8 771 773 10.1038/ncb1435 16733527 PMC5685802

[B11] Soboloff J Spassova MA Tang XD Hewavitharana T Xu W Gill DL Orai1 and STIM reconstitute store-operated calcium channel function J Biol Chem 2006 281 20661 20665 10.1074/jbc.C600126200 16766533

[B12] Mercer JC Dehaven WI Smyth JT Wedel B Boyles RR Bird GS Putney JW Jr Large store-operated calcium selective currents due to co-expression of Orai1 or Orai2 with the intracellular calcium sensor, Stim1 J Biol Chem 2006 281 24979 24990 10.1074/jbc.M604589200 16807233 PMC1633822

[B13] Hogan PG Lewis RS Rao A Molecular basis of calcium signaling in lymphocytes: STIM and ORAI Annu Rev Immunol 2010 28 491 533 10.1146/annurev.immunol.021908.132550 20307213 PMC2861828

[B14] Hogan PG Rao A Dissecting I_CRAC_, a store-operated calcium current Trends Biochem Sci 2007 32 235 245 10.1016/j.tibs.2007.03.009 17434311

[B15] Luik RM Wu MM Buchanan J Lewis RS The elementary unit of store-operated Ca^2+ ^entry: local activation of CRAC channels by STIM1 at ER-plasma membrane junctions J Cell Biol 2006 174 815 825 10.1083/jcb.200604015 16966423 PMC2064336

[B16] Wu MM Buchanan J Luik RM Lewis RS Ca^2+ ^store depletion causes STIM1 to accumulate in ER regions closely associated with the plasma membrane J Cell Biol 2006 174 803 813 10.1083/jcb.200604014 16966422 PMC2064335

[B17] Zhang SL Yu Y Roos J Kozak JA Deerinck TJ Ellisman MH Stauderman KA Cahalan MD STIM1 is a Ca^2+ ^sensor that activates CRAC channels and migrates from the Ca^2+ ^store to the plasma membrane Nature 2005 437 902 905 10.1038/nature04147 16208375 PMC1618826

[B18] Xu P Lu J Li Z Yu X Chen L Xu T Aggregation of STIM1 underneath the plasma membrane induces clustering of Orai1 Biochem Biophys Res Commun 2006 350 969 976 10.1016/j.bbrc.2006.09.134 17045966

[B19] Baba Y Hayashi K Fujii Y Mizushima A Watarai H Wakamori M Numaga T Mori Y Iino M Hikida M Kurosaki T Coupling of STIM1 to store-operated Ca^2+ ^entry through its constitutive and inducible movement in the endoplasmic reticulum Proc Natl Acad Sci USA 2006 103 16704 16709 10.1073/pnas.0608358103 17075073 PMC1636519

[B20] Liou J Fivaz M Inoue T Meyer T Live-cell imaging reveals sequential oligomerization and local plasma membrane targeting of stromal interaction molecule 1 after Ca^2+ ^store depletion Proc Natl Acad Sci USA 2007 104 9301 9306 10.1073/pnas.0702866104 17517596 PMC1890489

[B21] Luik RM Wang B Prakriya M Wu MM Lewis RS Oligomerization of STIM1 couples ER calcium depletion to CRAC channel activation Nature 2008 454 538 542 10.1038/nature07065 18596693 PMC2712442

[B22] Stathopulos PB Zheng L Li GY Plevin MJ Ikura M Structural and mechanistic insights into STIM1-mediated initiation of store-operated calcium entry Cell 2008 135 110 122 10.1016/j.cell.2008.08.006 18854159

[B23] Stathopulos PB Li GY Plevin MJ Ames JB Ikura M Stored Ca^2+ ^depletion-induced oligomerization of stromal interaction molecule 1 (STIM1) via the EF-SAM region: An initiation mechanism for capacitive Ca^2+ ^entry J Biol Chem 2006 281 35855 35862 10.1074/jbc.M608247200 17020874

[B24] Wu MM Luik RM Lewis RS Some assembly required: constructing the elementary units of store-operated Ca^2+ ^entry Cell Calcium 2007 42 163 172 10.1016/j.ceca.2007.03.003 17499354 PMC2323433

[B25] Huang GN Zeng W Kim JY Yuan JP Han L Muallem S Worley PF STIM1 carboxyl-terminus activates native SOC, I_CRAC _and TRPC1 channels Nat Cell Biol 2006 8 1003 1010 10.1038/ncb1454 16906149

[B26] Park CY Hoover PJ Mullins FM Bachhawat P Covington ED Raunser S Walz T Garcia KC Dolmetsch RE Lewis RS STIM1 clusters and activates CRAC channels via direct binding of a cytosolic domain to Orai1 Cell 2009 136 876 890 10.1016/j.cell.2009.02.014 19249086 PMC2670439

[B27] Walsh CM Chvanov M Haynes LP Petersen OH Tepikin AV Burgoyne RD Role of phosphoinositides in STIM1 dynamics and store-operated calcium entry Biochem J 2009 425 159 168 10.1042/BJ20090884 19843011 PMC2860680

[B28] Ercan E Momburg F Engel U Temmerman K Nickel W Seedorf M A conserved, lipid-mediated sorting mechanism of yeast Ist2 and mammalian STIM proteins to the peripheral ER Traffic 2009 10 1802 1818 10.1111/j.1600-0854.2009.00995.x 19845919

[B29] Li Z Lu J Xu P Xie X Chen L Xu T Mapping the interacting domains of STIM1 and Orai1 in Ca^2+ ^release-activated Ca^2+ ^channel activation J Biol Chem 2007 282 29448 29456 10.1074/jbc.M703573200 17702753

[B30] Muik M Frischauf I Derler I Fahrner M Bergsmann J Eder P Schindl R Hesch C Polzinger B Fritsch R Groschner K Romanin C Dynamic coupling of the putative coiled-coil domain of ORAI1 with STIM1 mediates ORAI1 channel activation J Biol Chem 2008 283 8014 8022 10.1074/jbc.M708898200 18187424

[B31] Zhou Y Meraner P Kwon HT Machnes D Oh-hora M Zimmer J Huang Y Stura A Rao A Hogan PG STIM1 gates the store-operated calcium channel ORAI1 in vitro Nat Struct Mol Biol 2010 17 112 116 10.1038/nsmb.1724 20037597 PMC2902271

[B32] Yuan JP Zeng W Dorwart MR Choi YJ Worley PF Muallem S SOAR and the polybasic STIM1 domains gate and regulate Orai channels Nat Cell Biol 2009 11 337 343 10.1038/ncb1842 19182790 PMC2663385

[B33] Muik M Fahrner M Derler I Schindl R Bergsmann J Frischauf I Groschner K Romanin C A cytosolic homomerization and a modulatory domain within STIM1 C terminus determine coupling to ORAI1 channels J Biol Chem 2009 284 8421 8426 10.1074/jbc.C800229200 19189966 PMC2659200

[B34] Kawasaki T Lange I Feske S A minimal regulatory domain in the C terminus of STIM1 binds to and activates ORAI1 CRAC channels Biochem Biophys Res Commun 2009 385 49 54 10.1016/j.bbrc.2009.05.020 19433061 PMC2821023

[B35] Feng M Grice DM Faddy HM Nguyen N Leitch S Wang Y Muend S Kenny PA Sukumar S Roberts-Thomson SJ Monteith GR Rao R Store-independent activation of Orai1 by SPCA2 in mammary tumors Cell 2010 143 84 98 10.1016/j.cell.2010.08.040 20887894 PMC2950964

[B36] Williams RT Manji SS Parker NJ Hancock MS Van Stekelenburg L Eid JP Senior PV Kazenwadel JS Shandala T Saint R Smith PJ Dziadek MA Identification and characterization of the STIM (stromal interaction molecule) gene family: coding for a novel class of transmembrane proteins Biochem J 2001 357 673 685 10.1042/0264-6021:3570673 11463338 PMC1221997

[B37] Brandman O Liou J Park WS Meyer T STIM2 is a feedback regulator that stabilizes basal cytosolic and endoplasmic reticulum Ca^2+ ^levels Cell 2007 131 1327 1339 10.1016/j.cell.2007.11.039 18160041 PMC2680164

[B38] Decuypere JP Monaco G Kiviluoto S Oh-hora M Luyten T De Smedt H Parys JB Missiaen L Bultynck G STIM1, but not STIM2, is required for proper agonist-induced Ca^2+ ^signaling Cell Calcium 2010 48 161 167 10.1016/j.ceca.2010.08.003 20801505

[B39] Oh-Hora M Yamashita M Hogan PG Sharma S Lamperti E Chung W Prakriya M Feske S Rao A Dual functions for the endoplasmic reticulum calcium sensors STIM1 and STIM2 in T cell activation and tolerance Nat Immunol 2008 9 432 443 10.1038/ni1574 18327260 PMC2737533

[B40] Feske S Prakriya M Rao A Lewis RS A severe defect in CRAC Ca^2+ ^channel activation and altered K^+ ^channel gating in T cells from immunodeficient patients J Exp Med 2005 202 651 662 10.1084/jem.20050687 16147976 PMC2212870

[B41] McCarl CA Picard C Khalil S Kawasaki T Rother J Papolos A Kutok J Hivroz C Ledeist F Plogmann K Ehl S Notheis G Albert MH Belohradsky BH Kirschner J Rao A Fischer A Feske S ORAI1 deficiency and lack of store-operated Ca^2+ ^entry cause immunodeficiency, myopathy, and ectodermal dysplasia J Allergy Clin Immunol 2009 124 1311 1318 e1317 10.1016/j.jaci.2009.10.007 20004786 PMC2829767

[B42] Picard C McCarl CA Papolos A Khalil S Luthy K Hivroz C LeDeist F Rieux-Laucat F Rechavi G Rao A Fischer A Feske S STIM1 mutation associated with a syndrome of immunodeficiency and autoimmunity N Engl J Med 2009 360 1971 1980 10.1056/NEJMoa0900082 19420366 PMC2851618

[B43] Feske S Draeger R Peter HH Eichmann K Rao A The duration of nuclear residence of NFAT determines the pattern of cytokine expression in human SCID T cells J Immunol 2000 165 297 305 10861065 10.4049/jimmunol.165.1.297

[B44] Feske S ORAI1 and STIM1 deficiency in human and mice: roles of store-operated Ca^2+ ^entry in the immune system and beyond Immunol Rev 2009 231 189 209 10.1111/j.1600-065X.2009.00818.x 19754898 PMC9011976

[B45] Grigoriev I Gouveia SM van der Vaart B Demmers J Smyth JT Honnappa S Splinter D Steinmetz MO Putney JW Jr Hoogenraad CC Akhmanova A STIM1 is a MT-plus-end-tracking protein involved in remodeling of the ER Curr Biol 2008 18 177 182 10.1016/j.cub.2007.12.050 18249114 PMC2600655

[B46] Honnappa S Gouveia SM Weisbrich A Damberger FF Bhavesh NS Jawhari H Grigoriev I van Rijssel FJ Buey RM Lawera A Jelesarov I Winkler FK Wüthrich K Akhmanova A Steinmetz MO An EB1-binding motif acts as a microtubule tip localization signal Cell 2009 138 366 376 10.1016/j.cell.2009.04.065 19632184

[B47] Bakowski D Glitsch MD Parekh AB An examination of the secretion-like coupling model for the activation of the Ca^2+ ^release-activated Ca^2+ ^current I_CRAC _in RBL-1 cells J Physiol 2001 532 55 71 10.1111/j.1469-7793.2001.0055g.x 11283225 PMC2278514

[B48] Quintana A Schwarz EC Schwindling C Lipp P Kaestner L Hoth M Sustained activity of calcium release-activated calcium channels requires translocation of mitochondria to the plasma membrane J Biol Chem 2006 281 40302 40309 10.1074/jbc.M607896200 17056596

[B49] Yuan JP Zeng W Huang GN Worley PF Muallem S STIM1 heteromultimerizes TRPC channels to determine their function as store-operated channels Nat Cell Biol 2007 9 636 645 10.1038/ncb1590 17486119 PMC2699187

[B50] Zeng W Yuan JP Kim MS Choi YJ Huang GN Worley PF Muallem S STIM1 gates TRPC channels, but not Orai1, by electrostatic interaction Mol Cell 2008 32 439 448 10.1016/j.molcel.2008.09.020 18995841 PMC2586614

[B51] DeHaven WI Jones BF Petranka JG Smyth JT Tomita T Bird GS Putney JW Jr TRPC channels function independently of STIM1 and Orai1 J Physiol 2009 587 2275 2298 10.1113/jphysiol.2009.170431 19332491 PMC2697298

[B52] Lee KP Yuan JP Hong JH So I Worley PF Muallem S An endoplasmic reticulum/plasma membrane junction: STIM1/Orai1/TRPCs FEBS Lett 2010 584 2022 2027 10.1016/j.febslet.2009.11.078 19944100 PMC2866752

[B53] Shuttleworth TJ Thompson JL Mignen O STIM1 and the noncapacitative ARC channels Cell Calcium 2007 42 183 191 10.1016/j.ceca.2007.01.012 17391754 PMC1995027

[B54] Mignen O Thompson JL Shuttleworth TJ STIM1 regulates Ca^2+ ^entry via arachidonate-regulated Ca^2+^-selective (ARC) channels without store depletion or translocation to the plasma membrane J Physiol 2007 579 703 715 10.1113/jphysiol.2006.122432 17158173 PMC2151373

[B55] Mignen O Thompson JL Shuttleworth TJ Both Orai1 and Orai3 are essential components of the arachidonate-regulated Ca^2+^-selective (ARC) channels J Physiol 2008 586 185 195 10.1113/jphysiol.2007.146258 17991693 PMC2375546

[B56] Lefkimmiatis K Srikanthan M Maiellaro I Moyer MP Curci S Hofer AM Store-operated cyclic AMP signalling mediated by STIM1 Nat Cell Biol 2009 11 433 442 10.1038/ncb1850 19287379

[B57] Roy J Lefkimmiatis K Moyer MP Curci S Hofer AM The {omega}-3 fatty acid eicosapentaenoic acid elicits cAMP generation in colonic epithelial cells via a "store-operated" mechanism Am J Physiol Gastrointest Liver Physiol 2010 299 G715 722 10.1152/ajpgi.00028.2010 20576916 PMC2950681

[B58] Wang Y Deng X Mancarella S Hendron E Eguchi S Soboloff J Tang XD Gill DL The calcium store sensor, STIM1, reciprocally controls Orai and CaV1.2 channels Science 2010 330 105 109 10.1126/science.1191086 20929813 PMC3601900

[B59] Park CY Shcheglovitov A Dolmetsch R The CRAC channel activator STIM1 binds and inhibits L-type voltage-gated calcium channels Science 2010 330 101 105 10.1126/science.1191027 20929812

[B60] Kotturi MF Carlow DA Lee JC Ziltener HJ Jefferies WA Identification and functional characterization of voltage-dependent calcium channels in T lymphocytes J Biol Chem 2003 278 46949 46960 10.1074/jbc.M309268200 12954628

[B61] Kotturi MF Jefferies WA Molecular characterization of L-type calcium channel splice variants expressed in human T lymphocytes Mol Immunol 2005 42 1461 1474 10.1016/j.molimm.2005.01.014 15899519

[B62] Lyfenko AD Dirksen RT Differential dependence of store-operated and excitation-coupled Ca^2+ ^entry in skeletal muscle on STIM1 and Orai1 J Physiol 2008 586 4815 4824 10.1113/jphysiol.2008.160481 18772199 PMC2614059

[B63] Stiber J Hawkins A Zhang ZS Wang S Burch J Graham V Ward CC Seth M Finch E Malouf N Williams RS Eu JP Rosenberg P STIM1 signalling controls store-operated calcium entry required for development and contractile function in skeletal muscle Nat Cell Biol 2008 10 688 697 10.1038/ncb1731 18488020 PMC2694045

[B64] Wang Y Deng X Hewavitharana T Soboloff J Gill DL Stim, ORAI and TRPC channels in the control of calcium entry signals in smooth muscle Clin Exp Pharmacol Physiol 2008 35 1127 1133 10.1111/j.1440-1681.2008.05018.x 18782202 PMC3601895

[B65] Potier M Gonzalez JC Motiani RK Abdullaev IF Bisaillon JM Singer HA Trebak M Evidence for STIM1- and Orai1-dependent store-operated calcium influx through I_CRAC _in vascular smooth muscle cells: role in proliferation and migration FASEB J 2009 23 2425 2437 10.1096/fj.09-131128 19364762 PMC2717784

[B66] Berna-Erro A Braun A Kraft R Kleinschnitz C Schuhmann MK Stegner D Wultsch T Eilers J Meuth SG Stoll G Nieswandt B STIM2 regulates capacitive Ca^2+ ^entry in neurons and plays a key role in hypoxic neuronal cell death Sci Signal 2009 2 ra67 10.1126/scisignal.2000522 19843959

[B67] Wu C Orozco C Boyer J Leglise M Goodale J Batalov S Hodge C Haase J Janes J Huss J Su A BioGPS: an extensible and customizable portal for querying and organizing gene annotation resources Genome Biol 2009 10 R130 10.1186/gb-2009-10-11-r130 19919682 PMC3091323

[B68] Lattin J Schroder K Su A Walker J Zhang J Wiltshire T Saijo K Glass C Hume D Kellie S Sweet M Expression analysis of G Protein-Coupled Receptors in mouse macrophages Immunome Res 2008 4 5 10.1186/1745-7580-4-5 18442421 PMC2394514

[B69] Kurebayashi N Ogawa Y Depletion of Ca^2+ ^in the sarcoplasmic reticulum stimulates Ca^2+ ^entry into mouse skeletal muscle fibres J Physiol 2001 533 185 199 10.1111/j.1469-7793.2001.0185b.x 11351027 PMC2278591

[B70] Pan Z Yang D Nagaraj RY Nosek TA Nishi M Takeshima H Cheng H Ma J Dysfunction of store-operated calcium channel in muscle cells lacking mg29 Nat Cell Biol 2002 4 379 383 10.1038/ncb788 11988740

[B71] Rosenberg P Hawkins A Stiber J Shelton JM Hutcheson K Bassel-Duby R Shin DM Yan Z Williams RS TRPC3 channels confer cellular memory of recent neuromuscular activity Proc Natl Acad Sci USA 2004 101 9387 9392 10.1073/pnas.0308179101 15199180 PMC438986

[B72] Darbellay B Arnaudeau S Konig S Jousset H Bader C Demaurex N Bernheim L STIM1- and Orai1-dependent store-operated calcium entry regulates human myoblast differentiation J Biol Chem 2009 284 5370 5380 10.1074/jbc.M806726200 19088073

[B73] Vandebrouck C Martin D Colson-Van Schoor M Debaix H Gailly P Involvement of TRPC in the abnormal calcium influx observed in dystrophic (mdx) mouse skeletal muscle fibers J Cell Biol 2002 158 1089 1096 10.1083/jcb.200203091 12235126 PMC2173225

[B74] Vandebrouck A Ducret T Basset O Sebille S Raymond G Ruegg U Gailly P Cognard C Constantin B Regulation of store-operated calcium entries and mitochondrial uptake by minidystrophin expression in cultured myotubes FASEB J 2006 20 136 138 16254044 10.1096/fj.04-3633fje

[B75] Millay DP Goonasekera SA Sargent MA Maillet M Aronow BJ Molkentin JD Calcium influx is sufficient to induce muscular dystrophy through a TRPC-dependent mechanism Proc Natl Acad Sci USA 2009 106 19023 19028 10.1073/pnas.0906591106 19864620 PMC2776441

[B76] Dirksen RT Checking your SOCCs and feet: the molecular mechanisms of Ca^2+ ^entry in skeletal muscle J Physiol 2009 587 3139 3147 10.1113/jphysiol.2009.172148 19406875 PMC2727026

[B77] Launikonis BS Murphy RM Edwards JN Toward the roles of store-operated Ca^2+ ^entry in skeletal muscle Pflugers Arch 2010 460 813 823 10.1007/s00424-010-0856-7 20577885

[B78] Kiselyov KI Shin DM Wang Y Pessah IN Allen PD Muallem S Gating of store-operated channels by conformational coupling to ryanodine receptors Mol Cell 2000 6 421 431 10.1016/S1097-2765(00)00041-1 10983988

[B79] Lee EH Cherednichenko G Pessah IN Allen PD Functional coupling between TRPC3 and RyR1 regulates the expressions of key triadic proteins J Biol Chem 2006 281 10042 10048 10.1074/jbc.M600981200 16484216

[B80] Launikonis BS Barnes M Stephenson DG Identification of the coupling between skeletal muscle store-operated Ca^2+ ^entry and the inositol trisphosphate receptor Proc Natl Acad Sci USA 2003 100 2941 2944 10.1073/pnas.0536227100 12601149 PMC151445

[B81] Estrada M Espinosa A Gibson CJ Uhlen P Jaimovich E Capacitative calcium entry in testosterone-induced intracellular calcium oscillations in myotubes J Endocrinol 2005 184 371 379 10.1677/joe.1.05921 15684345

[B82] Kiselyov K Xu X Mozhayeva G Kuo T Pessah I Mignery G Zhu X Birnbaumer L Muallem S Functional interaction between InsP3 receptors and store-operated Htrp3 channels Nature 1998 396 478 482 10.1038/24890 9853757

[B83] Powell JA Carrasco MA Adams DS Drouet B Rios J Muller M Estrada M Jaimovich E IP_3 _receptor function and localization in myotubes: an unexplored Ca^2+ ^signaling pathway in skeletal muscle J Cell Sci 2001 114 3673 3683 11707519 10.1242/jcs.114.20.3673

[B84] Cardenas C Liberona JL Molgo J Colasante C Mignery GA Jaimovich E Nuclear inositol 1,4,5-trisphosphate receptors regulate local Ca^2+ ^transients and modulate cAMP response element binding protein phosphorylation J Cell Sci 2005 118 3131 3140 10.1242/jcs.02446 16014380

[B85] Vig M DeHaven WI Bird GS Billingsley JM Wang H Rao PE Hutchings AB Jouvin MH Putney JW Kinet JP Defective mast cell effector functions in mice lacking the CRACM1 pore subunit of store-operated calcium release-activated calcium channels Nat Immunol 2008 9 89 96 10.1038/ni1550 18059270 PMC2377025

[B86] Smyth JT Dehaven WI Bird GS Putney JW Jr Ca^2+^-store-dependent and -independent reversal of Stim1 localization and function J Cell Sci 2008 121 762 772 10.1242/jcs.023903 18285445 PMC2587154

[B87] Louis M Zanou N Van Schoor M Gailly P TRPC1 regulates skeletal myoblast migration and differentiation J Cell Sci 2008 121 3951 3959 10.1242/jcs.037218 19001499

[B88] Zanou Ng Shapovalov G Louis M Tajeddine N Gallo C Van Schoor M Anguish I Cao ML Schakman O Dietrich A Lebacq J Ruegg U Roulet E Birnbaumer L Gailly P Role of TRPC1 channel in skeletal muscle function Am J Physiol - Cell Physiol 2010 298 C149 162 10.1152/ajpcell.00241.2009 PMC2806157 19846750

[B89] Chen CMA Kraut N Groudine M Weintraub H I-mf, a novel myogenic repressor, interacts with members of the MyoD family Cell 1996 86 731 741 10.1016/S0092-8674(00)80148-8 8797820

[B90] Ma R Rundle D Jacks J Koch M Downs T Tsiokas L Inhibitor of myogenic family, a novel suppressor of store-operated currents through an interaction with TRPC1 J Biol Chem 2003 278 52763 52772 10.1074/jbc.M309610200 14530267

[B91] Launikonis BS Rios E Store-operated Ca^2+ ^entry during intracellular Ca^2+ ^release in mammalian skeletal muscle J Physiol 2007 583 81 97 10.1113/jphysiol.2007.135046 17569733 PMC2277221

[B92] Edwards JN Murphy RM Cully TR von Wegner F Friedrich O Launikonis BS Ultra-rapid activation and deactivation of store-operated Ca^2+ ^entry in skeletal muscle Cell Calcium 2010 47 458 467 10.1016/j.ceca.2010.04.001 20434768

[B93] Hirata Y Brotto M Weisleder N Chu Y Lin P Zhao X Thornton A Komazaki S Takeshima H Ma J Pan Z Uncoupling store-operated Ca^2+ ^entry and altered Ca^2+ ^release from sarcoplasmic reticulum through silencing of junctophilin genes Biophys J 2006 90 4418 4427 10.1529/biophysj.105.076570 16565048 PMC1471867

[B94] Landstrom AP Weisleder N Batalden KB Martijn Bos J Tester DJ Ommen SR Wehrens XHT Claycomb WC Ko JK Hwang M Pan Z Ma J Ackerman MJ Mutations in JPH2-encoded junctophilin-2 associated with hypertrophic cardiomyopathy in humans J Mol Cell Cardiol 2007 42 1026 1035 10.1016/j.yjmcc.2007.04.006 17509612 PMC4318564

[B95] Woo JS Hwang JH Ko JK Weisleder N Kim DH Ma J Lee EH S165F mutation of junctophilin 2 affects Ca^2+ ^signalling in skeletal muscle Biochem J 2010 427 125 134 10.1042/BJ20091225 20095964

[B96] Li H Ding X Lopez JR Takeshima H Ma J Allen PD Eltit JM Impaired Orai1-mediated resting Ca^2+ ^entry reduces the cytosolic [Ca^2+^] and sarcoplasmic reticulum Ca^2+ ^loading in quiescent junctophilin 1 knock-out myotubes J Biol Chem 2010 285 39171 39179 10.1074/jbc.M110.149690 20937810 PMC2998103

[B97] Shin DM Muallem S Skeletal muscle dressed in SOCs Nat Cell Biol 2008 10 639 641 10.1038/ncb0608-639 18521070

[B98] De Smedt H Parys JB Himpens B Missiaen L Borghgraef R Changes in the mechanism of Ca^2+ ^mobilization during the differentiation of BC3H1 muscle cells Biochem J 1991 273 219 223 1989585 10.1042/bj2730219 PMC1149902

[B99] Arai M Otsu K MacLennan DH Periasamy M Regulation of sarcoplasmic reticulum gene expression during cardiac and skeletal muscle development Am J Physiol 1992 262 C614 620 1372478 10.1152/ajpcell.1992.262.3.C614

[B100] Friday BB Horsley V Pavlath GK Calcineurin activity is required for the initiation of skeletal muscle differentiation J Cell Biol 2000 149 657 666 10.1083/jcb.149.3.657 10791979 PMC2174840

[B101] Kegley KM Gephart J Warren GL Pavlath GK Altered primary myogenesis in *NFATC3*^*-/- *^mice leads to decreased muscle size in the adult Dev Biol 2001 232 115 126 10.1006/dbio.2001.0179 11254352

[B102] Boittin FX Petermann O Hirn C Mittaud P Dorchies OM Roulet E Ruegg UT Ca^2+^-independent phospholipase A2 enhances store-operated Ca^2+ ^entry in dystrophic skeletal muscle fibers J Cell Sci 2006 119 3733 3742 10.1242/jcs.03184 16926189

[B103] Allen DG Gervasio OL Yeung EW Whitehead NP Calcium and the damage pathways in muscular dystrophy Can J Physiol Pharmacol 2010 88 83 91 10.1139/Y09-058 20237582

[B104] Vandebrouck A Sabourin J Rivet J Balghi H Sebille S Kitzis A Raymond G Cognard C Bourmeyster N Constantin B Regulation of capacitative calcium entries by alpha1-syntrophin: association of TRPC1 with dystrophin complex and the PDZ domain of alpha1-syntrophin FASEB J 2007 21 608 617 10.1096/fj.06-6683com 17202249

[B105] Sabourin J Lamiche C Vandebrouck A Magaud C Rivet J Cognard C Bourmeyster N Constantin B Regulation of TRPC1 and TRPC4 cation channels requires an alpha1-syntrophin-dependent complex in skeletal mouse myotubes J Biol Chem 2009 284 36248 36261 10.1074/jbc.M109.012872 19812031 PMC2794741

[B106] Edwards JN Friedrich O Cully TR von Wegner F Murphy RM Launikonis BS Upregulation of store-operated Ca^2+ ^entry in dystrophic mdx mouse muscle Am J Physiol Cell Physiol 2010 299 C42 50 10.1152/ajpcell.00524.2009 20427714

[B107] Yeung EW Whitehead NP Suchyna TM Gottlieb PA Sachs F Allen DG Effects of stretch-activated channel blockers on [Ca^2+^]_i _and muscle damage in the mdx mouse J Physiol 2005 562 367 380 10.1113/jphysiol.2004.075275 15528244 PMC1665499

[B108] Basset O Boittin FX Cognard C Constantin B Ruegg UT Bcl-2 overexpression prevents calcium overload and subsequent apoptosis in dystrophic myotubes Biochem J 2006 395 267 276 10.1042/BJ20051265 16393138 PMC1422769

[B109] Rong YP Aromolaran AS Bultynck G Zhong F Li X McColl K Matsuyama S Herlitze S Roderick HL Bootman MD Mignery GA Parys JB De Smedt H Distelhorst CW Targeting Bcl-2-IP_3 _receptor interaction to reverse Bcl-2's inhibition of apoptotic calcium signals Mol Cell 2008 31 255 265 10.1016/j.molcel.2008.06.014 18657507 PMC3660092

[B110] Rong YP Bultynck G Aromolaran AS Zhong F Parys JB De Smedt H Mignery GA Roderick HL Bootman MD Distelhorst CW The BH4 domain of Bcl-2 inhibits ER calcium release and apoptosis by binding the regulatory and coupling domain of the IP_3 _receptor Proc Natl Acad Sci USA 2009 106 14397 14402 10.1073/pnas.0907555106 19706527 PMC2728114

[B111] Stiber JA Zhang ZS Burch J Eu JP Zhang S Truskey GA Seth M Yamaguchi N Meissner G Shah R Worley PF Williams RS Rosenberg PB Mice lacking Homer 1 exhibit a skeletal myopathy characterized by abnormal transient receptor potential channel activity Mol Cell Biol 2008 28 2637 2647 10.1128/MCB.01601-07 18268005 PMC2293116

[B112] Cardenas C Juretic N Bevilacqua JA Garcia IE Figueroa R Hartley R Taratuto AL Gejman R Riveros N Molgō J Jaimovich E Abnormal distribution of inositol 1,4,5-trisphosphate receptors in human muscle can be related to altered calcium signals and gene expression in Duchenne dystrophy-derived cells FASEB J 2010 24 3210 3221 10.1096/fj.09-152017 20395455

[B113] Bultynck G De Smet P Rossi D Callewaert G Missiaen L Sorrentino V De Smedt H Parys JB Characterization and mapping of the 12 kDa FK506-binding protein (FKBP12)-binding site on different isoforms of the ryanodine receptor and of the inositol 1,4,5-trisphosphate receptor Biochem J 2001 354 413 422 10.1042/0264-6021:3540413 11171121 PMC1221670

[B114] Vangheluwe P Schuermans M Zādor E Waelkens E Raeymaekers L Wuytack F Sarcolipin and phospholamban mRNA and protein expression in cardiac and skeletal muscle of different species Biochem J 2005 389 151 159 10.1042/BJ20050068 15801907 PMC1184547

[B115] Parys JB de Smedt H Missiaen L Bootman MD Sienaert I Casteels R Rat basophilic leukemia cells as model system for inositol 1,4,5-trisphosphate receptor IV, a receptor of the type II family: functional comparison and immunological detection Cell Calcium 1995 17 239 249 10.1016/0143-4160(95)90070-5 7664312

[B116] Edgar R Domrachev M Lash AE Gene Expression Omnibus: NCBI gene expression and hybridization array data repository Nucleic Acids Res 2002 30 207 210 10.1093/nar/30.1.207 11752295 PMC99122

[B117] Haslett J Sanoudou D Kho A Han M Bennett R Kohane I Beggs A Kunkel L Gene expression profiling of Duchenne muscular dystrophy skeletal muscle Neurogenetics 2003 4 163 171 10.1007/s10048-003-0148-x 12698323

[B118] Haslett JN Sanoudou D Kho AT Bennett RR Greenberg SA Kohane IS Beggs AH Kunkel LM Gene expression comparison of biopsies from Duchenne muscular dystrophy (DMD) and normal skeletal muscle Proc Natl Acad Sci USA 2002 99 15000 15005 10.1073/pnas.192571199 12415109 PMC137534

